# Negative Pressure Wound Therapy Decreases Mortality in a Murine Model of Burn-Wound Sepsis Involving *Pseudomonas aeruginosa* Infection

**DOI:** 10.1371/journal.pone.0090494

**Published:** 2014-02-28

**Authors:** Yang Liu, Qin Zhou, Yunchuan Wang, Zhengcai Liu, Maolong Dong, Yaojun Wang, Xiao Li, Dahai Hu

**Affiliations:** 1 Department of Burns and Cutaneous Surgery, Xijing Hospital, Fourth Military Medical University, Xi’an, Shaanxi Province, China; 2 Department of Hepatobiliary Surgery, Xijing Hospital, Fourth Military Medical University, Xi’an, Shaanxi Province, China; University of Cincinnati, United States of America

## Abstract

**Background:**

The colonization of burn wounds by *Pseudomonas aeruginosa* can lead to septic shock, organ injuries, and high mortality rates. We hypothesized that negative pressure wound therapy (NPWT) would decrease invasion and proliferation of *P. aeruginosa* within the burn wound and reduce mortality.

**Methods:**

Thermal injuries were induced in anesthetized mice, and *P. aeruginosa* was applied to the wound surface for 24 h. After removing the burn eschar and debridement, the animals were subjected to either NPWT or wet-to-dry (WTD) treatment protocols. The bacterial loads on the wound surface were assessed during 7 d of treatment, as were the concentrations of inflammatory cytokines in the peripheral blood samples. Survival was monitored daily for 14 d after burn induction. Finally, samples of wounded skin, lung, liver, and kidney were collected and subjected to histopathological examination.

**Results:**

Applying *P. aeruginosa* to the burn wound surface led to sepsis. During early stages of treatment, NPWT reduced the mortality of the septic animals and levels of *P. aeruginosa* within the burn wound compared with WTD-treated animals. Circulating levels of cytokines and cytoarchitectural abnormalities were also significantly reduced *via* NPWT.

**Conclusions:**

Our data indicate that NPWT inhibits the invasion and proliferation of *P. aeruginosa* in burn-wounded tissue and decreases early mortality in a murine model of burn-wound sepsis. These therapeutic benefits likely result from the ability of NPWT to decrease bacterial proliferation on the wound surface, reduce cytokine serum concentrations, and prevent damage to internal organs.

## Introduction

For patients with burn wounds, *Pseudomonas aeruginosa* (*P. aeruginosa*) is often the etiological agent of serious infection [1]. Acute burn wounds breach the protective barrier of the skin and suppress the immune system, both of which render the patient susceptible to bacterial infection. Once the wound has been colonized with *P. aeruginosa*, this bacterium rapidly proliferates within the damaged tissue. This event has high mortality rates and often leads to a disseminated infection, which can result in bacteremia, and septic shock [2]. The treatment of these infections is confounded by the resistance (both innate and acquired) of *P. aeruginosa* to many antimicrobials [3]. It is estimated that ∼50% of all deaths caused by burns result from infection, and untreatable forms of *P. aeruginosa* infection have unfortunately become more common [4]. It was initially postulated that the systemic dissemination of bacteria from the wound represents the source of sepsis [5]. Conventional wet-to-dry (WTD) treatment uses a saline dressing inside and dry dressing outside. WTD treatment keeps the wound moist and helps drain wound secretions. However, WTD is not particularly effective in clearing *P. aeruginosa* from the burn wound [6]. Many studies have demonstrated that *P. aeruginosa* rapidly proliferates within burned tissue, eventually spreading throughout the body *via* the circulatory system [5], [7]–[9]. This process induces uncontrolled inflammation, sepsis, and multi-organ failure.

Negative pressure wound therapy (NPWT) is commonly used to treat wounds, as it effectively clears bacteria from the wound [10]. A recent study indicated that NPWT significantly decreases the bacterial load of *P. aeruginosa* within a contaminated open fracture wound [11]. It is unclear, however, whether NPWT can effectively prevent sepsis when burn wounds are infected with *P. aeruginosa*.

We hypothesized that NPWT would decrease the levels of *P. aeruginosa* within a burn wound, prevent sepsis, and reduce the mortality rate. To test this hypothesis, a murine model of *P. aeruginosa*–induced burn wound sepsis was established according to our previous report [12]. The effect of NPWT on the bacterial load on the wound surface was determined using an *in vivo* imaging system and direct culturing of the wound tissue. Mortality rates were also examined. We further speculated that NPWT would reduce the levels of Th1-type cytokines in the bloodstream and thereby protect the internal organs against infection.

## Materials and Methods

### Ethics Statements

All animal experiments were performed in accordance with the guidelines from the Administration of Animal Experiments for Medical Research Purposes issued by the Ministry of Health of China. The protocol was approved by the Animal Experiment Administration Committee of Fourth Military Medical University. All surgical procedures were performed under sodium pentobarbital anesthesia and in a clean surgical room with sterilized instruments. All efforts were made to minimize the suffering of the mice during the experiments.

### Mice

Eight- to 12-week-old male C57BL/6 mice were obtained from the Experimental Animal Center of The Fourth Military Medical University and housed in specific pathogen-free conditions. A total of 342 mice were involved in our experiments. Because each test was independently repeated three times, 6 mice were used for the detection of bioluminescent bacteria in the wound, 36 mice were used for the bacterial cultures, and 180 mice were used for the cytokine analysis. In addition, 120 mice were used for the mortality analysis and were randomly divided into a NPWT group, WTD group, scald with infection group, or scald without infection group (30 mice per group).

### Bacterial Strain and Culture Conditions

The *P. aeruginosa* strain was derived from the PAO1 parental strain. This bacterium was genetically engineered to fluoresce by random chromosomal insertion of the modified Photorhabdus luminescens luxCDABE operon [13]. The bacteria were grown overnight in Luria-Bertani medium at 37°C with shaking (120 rpm), which produced cultures with an optical density (600 nm) of 0.8. This corresponded to a bacterial density of 1×10^9^ colony-forming units (CFU)/ml.

### Animal Model of Thermal Injury and Infection

The murine model of sepsis that was originally developed by Yoav Barnea and Yehuda Barnea [14] was adopted for this study with slight modifications. All procedures were performed in a laboratory accredited by the Association for Assessment and Accreditation of Laboratory Animal Care, and the protocol was approved by the Institutional Animal Care and Use Committee. The mice were securely placed into a template with a round opening (2 cm in diameter) that exposed their shaved backs. A thermal injury was induced by exposing the shaved area of the skin to water vapor (100°C) for 8 s. This injury is nonlethal but results in a third-degree (full-thickness) burn to ∼6% of the animal’s body surface. Fluid replacement therapy, which consisted of an intraperitoneal injection of 30 ml/kg of 0.9% physiological saline, was administered immediately following the burn. The mice in the infection groups had 50 µl of the bacterial inoculums (1.0×10^9 ^cfu/ml) directly applied to the eschar. During recovery, the mice were housed under warming lights and observed frequently.

### Wound Treatment and Bacterial Measurements

The bioluminescence of the bacteria in the wound was detected using the IVIS 100 system (Xenogen Corporation/Caliper Life Sciences, Alameda, CA, USA). The animals were anesthetized 24 h after bacterial inoculation, and the burn eschar was removed under aseptic conditions. The wound was then washed twice with sterile 0.9% physiological saline to clear the wound secretions; the wound was then imaged. This process determined the baseline quantity of bacteria. Dressings were then applied to the wounds. The wounds were dressed using either conventional WTD or the V.A.C. System (KCI, San Antonio, TX, USA). The V.A.C. System used GranuFoam Dressing (KCI) and a continuous negative pressure of –125 mmHg. The wounds were debrided and irrigated every 24 h. The wounds were imaged after 0, 1, 3, 5, and 7 d. After the images were collected on day 7, the animals were euthanized, and tissue specimens were collected. Measurements were performed using the IVIS 100 system with a 1-min exposure. A binning of four images was used to increase the sensitivity (i.e., improve the signal-to-noise ratio) without compromising spatial resolution. The images were quantitatively analyzed using Living Image software, version 3.0 (Xenogen).

### Bacterial Cultures

After sterile saline solution was used to remove the surface exudates, a biopsy (i.e., viable tissue from the center of the wound) was collected under aseptic conditions using a scalpel. These tissue specimens were collected after 0, 1, 2, 3, 5, and 7 d. Three different points of wound tissues were extracted (for a total of approximately 1 g) from each mouse under sterile conditions. The tissues from each mouse were then ground in a mortar and mixed with an equivalent amount of 0.9% saline. Each specimen was weighed and homogenized under sterile conditions. The tissue solutions were then diluted 1,000 fold and seeded on the medium. Each homogenate was then cultured for 24 h to determine the amount of bacteria (i.e., CFU) within 1 g of the tissue. The biopsies were processed and evaluated blindly by a medical microbiologist.

### Mortality Analysis

Mortality was monitored daily for 14 d after burn induction, and the observation intervals were 24 h.

### Cytokine Analysis

Blood samples were drawn from the tail vein and allowed to clot. The samples were then centrifuged at 7,000×*g* for 10 min at 4°C using an IEC-Centra-8R centrifuge (Fisher Scientific Company, Pittsburgh, PA). The serum supernatants were then frozen at –80°C. After the samples were thawed, the cytokines were quantified in duplicate using commercially available enzyme-linked immunosorbent assay (ELISA) kits for IL-1β, IL-4, IL-6, IL-10, and tumor necrosis factor (TNF-α; BD Bioscience, San Diego, CA, USA).

### Histopathological Preparations

After 3 d of treatment, tissue samples from the wounded skin, lung, liver, and kidney were collected and washed repeatedly with physiological saline. All tissue samples were fixed in 10% buffered formalin. The samples were embedded in paraffin, and standard 5-µm sections were cut and stained with hematoxylin and eosin (HE). Each sample was then subjected to randomized and unbiased quantitative histometry analysis. High-resolution photomicrographs were captured at random locations within the sampling area of each section and analyzed by an experienced pathologist. The tissues were primarily examined for leukocyte infiltration and pathological changes to the tissue structure. For each HE staining slice, leukocyte accumulation areas were chosen and observed under a magnification of 400x. A total of 20 random fields on each slice were chosen. The amount of total leukocytes in the 20 visualized areas were calculated and scored according to the method used by Kubiak [15].

### Statistical Analyses

The results are presented as the mean ± standard error of the mean. Significant differences between groups were determined using either the Student’s *t*-test or an analysis of variance. The survival rates were calculated using the Kaplan-Meier method, and significant differences between groups were determined using the log-rank test. A two-tailed *p*-value <0.05 was considered significant. The statistical analyses were performed using SPSS 14.0 software.

## Results

### Bacterial Bioluminescence

**Figure 1:**
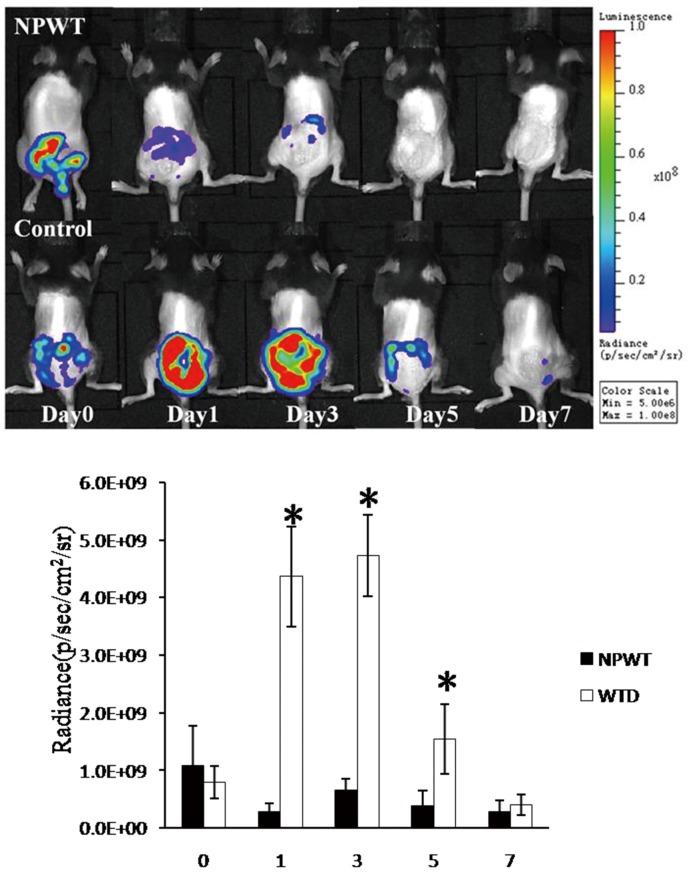
The burn wound bacteria levels following infection with bioluminescent *P. aeruginosa*. Comparisons are shown between NPWT and WTD (i.e., control) groups of mice during 7 d of treatment.

At 24 h after the wounds were infected with *P. aeruginosa*, baseline quantities of bacteria were determined. For the NPWT and WTD groups, the bacteria levels were 1.1×10^9^±6.8×10^8^ and 7.9×10^8^±2.8×10^8^ CFU, respectively (Figure 1, day 0). As such, the baseline bacteria levels were indistinguishable between the two groups (*t = *0.80, *p* = 0.22). During the early stages of infection (i.e., 24 and 72 h of treatment), however, significantly more bacteria were present in the wounds of the WTD group. For example, at 24 h, the NPWT and WTD groups had 3×10^8^±2×10^8^ and 4×10^9^±1×10^9^ CFU of bacteria, respectively (Figure 1, day 1; *t = *9.21, *p*<0.001). After 5 and 7 d of treatment, the bacterial counts were once again similar in the two groups. At day 5, the bacterial levels for the NPWT and WTD groups were 4×10^8^±6×10^8^ and 2×10^9^±2×10^9^ CFU, respectively (*t = *1.37, *p* = 0.11). During the late stages of infection, therefore, the NPWT and WTD treatments were equally effective in fighting infection.

### Bacterial Cultures of Wound Biopsies

**Figure 2:**
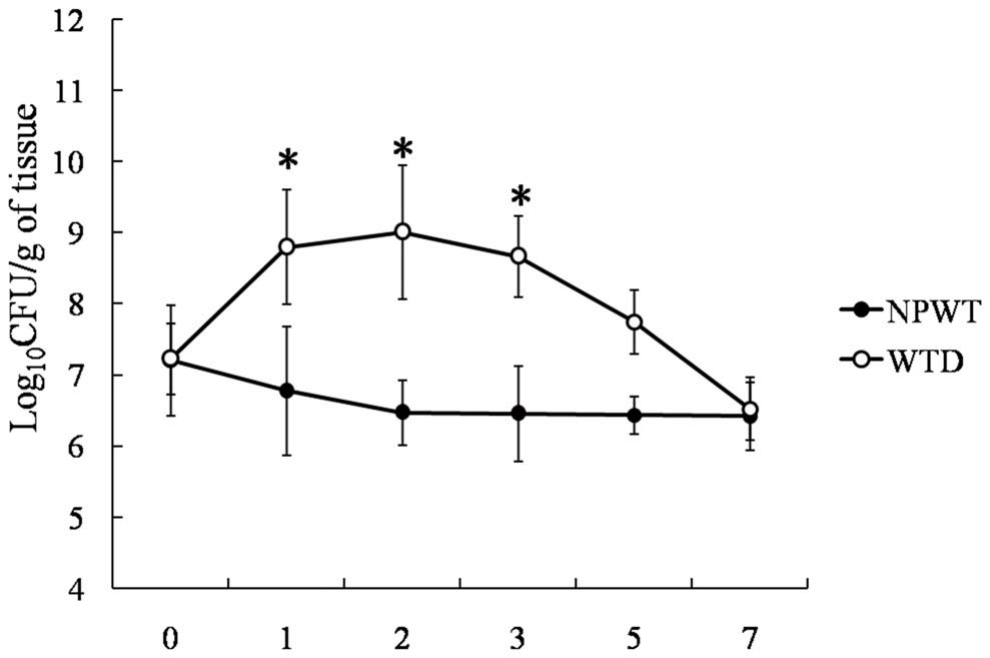
Bacterial cultures of wound biopsies collected from the NPWT and WTD groups.

*P. aeruginosa* was found in the cultures of all wound-tissue biopsies collected from the NPWT and WTD groups during the course of the experiment. Bacterial counts prior to treatment (day 0) demonstrated that the initial bacterial loads were similar among the NPWT and WTD groups (7.09±0.85 *vs.* 7.12±0.59 Log_10_ CFU/g; *p*>0.05). This result was in agreement with the bioluminescence data (Figure 1), although the CFU values were lower using this technique. On days 1, 2, 3, and 5 of the experiment, the bacteria levels within the wounds of the WTD animals were significantly higher than in those of the NPWT animals. At 7 d, however, the bacterial loads of the NPWT and WTD animals were indistinguishable (6.52±0.28 *vs.* 6.64±0.26 Log_10_ CFU/g; *p*>0.05). The results from bacterial cultures indicated, therefore, that the bacterial load was generally elevated in the wounds of WTD animals compared with the NPWT group. By day 7, however, the two treatments yielded similar results (Figure 2).

### Analysis of Cytokine Levels

**Figure 3:**
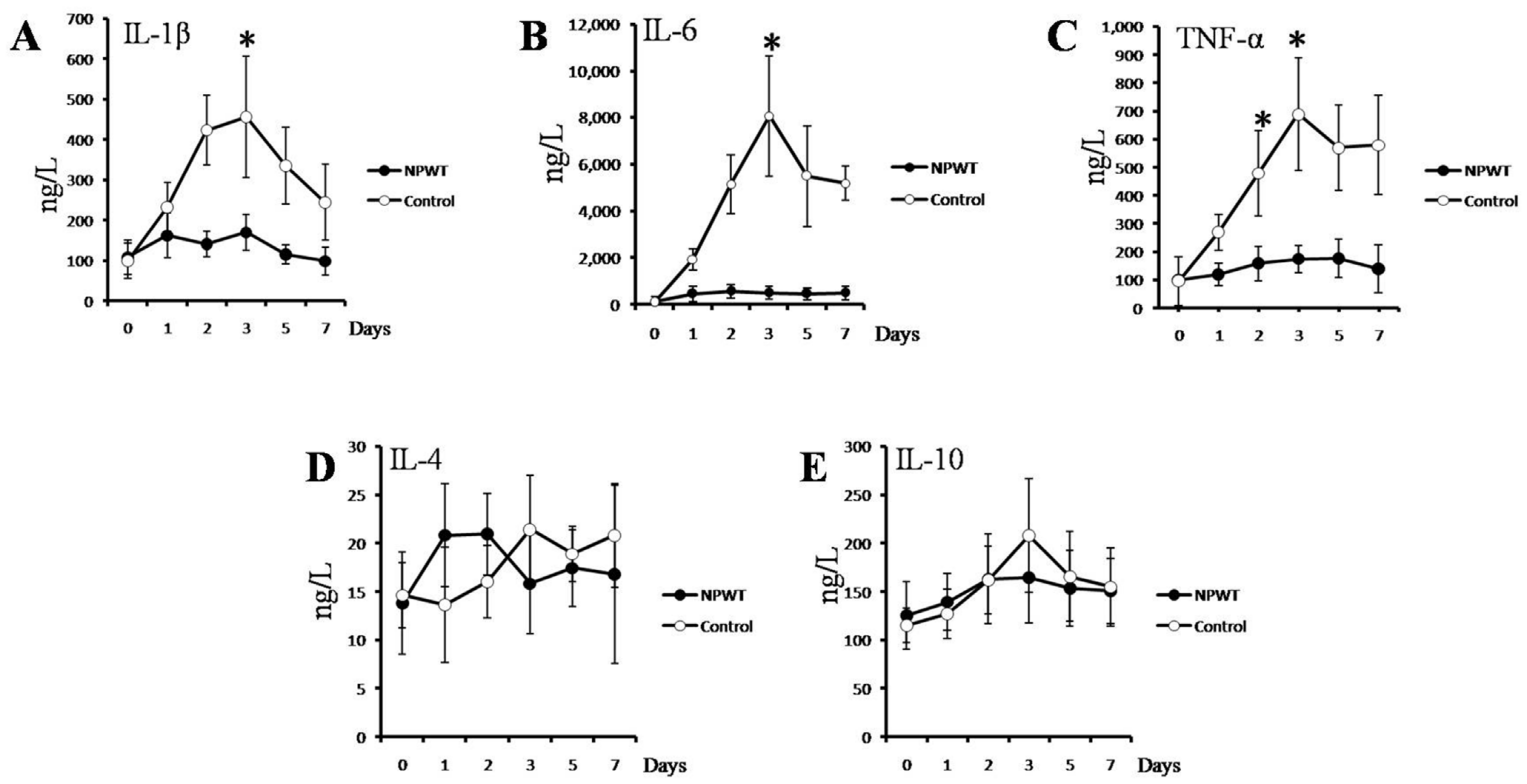
The levels of serum cytokines following burn-wound infection with *P. aeruginosa*. The data from 7(control) treatment are shown. The analyzed cytokines included (A) IL-1β, (B) IL-6, (C) TNF-α, (D) IL-4, and (E) IL-10. (A–C) The cytokine levels were generally elevated in the mice treated with WTD, reaching peak levels at 3 d post-infection. In contrast, the cytokine levels were not significantly elevated in the NPWT group. (D, E) For IL-4 and Il-10, significant deviations from baseline were not detected in either treatment group.

ELISA kits were used to measure the cytokine levels within the serum. The analyzed cytokines included IL-1β, IL-4, IL-6, IL-10, and TNF-α. For each measured cytokine, the baseline concentrations (day 0) indicated similar levels in the NPWT and WTD groups (*p*>0.05). Throughout the course of the experiment (i.e., 7 d of treatment), IL-1β levels did not significantly deviate from baseline in the NPWT group (*p*>0.05). In contrast, WTD-treated animals had dramatically elevated levels of IL-1β relative to baseline within 2 d (Figure 3A, B). When the IL-6 levels were analyzed, this cytokine was significantly elevated in the NPWT group, with peak values measured on day 3. No significant changes in IL-6 levels were detected relative to baseline for the WTD group (*p*>0.05; Figure 3C).

As treatment was initiated, TNF-α levels increased rapidly in the WTD group, reaching peak values after 3 d. No significant changes in TNF-α levels were detected in the NPWT group.

For both IL-4 and IL-10, no significant deviations from baseline were detected during 7 d of treatment in either the WTD or NPWT groups.

### Effect of NPWT Treatment on Survival

**Figure 4:**
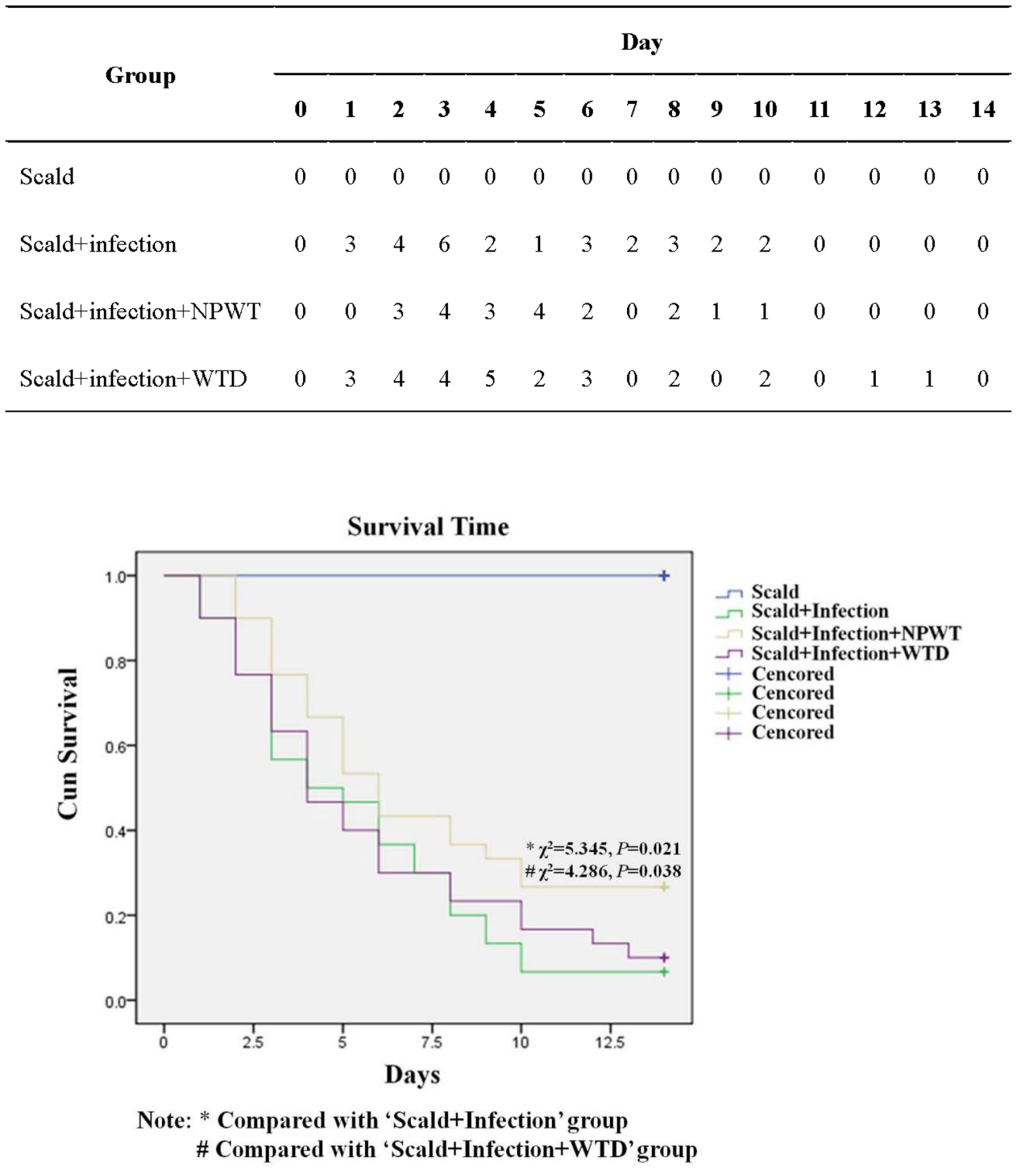
Survival rate analysis. NPWT resulted in a significantly higher survival rate than was observed in the scald plus infection group (*p*<0.01). A significant difference in survival was not detected between the groups treated with NPWT or WTD (*p*>0.05).

To test whether NPWT therapy could affect survival after burn, the wounds were infected with *P. aeruginosa*, and the animals were divided into four groups: scald, scald plus infection, scald plus infection plus NPWT, and scald plus infection plus WTD. Following injury, the 14-d survival rates were analyzed (Figure 4). After 14 d, 100% of the animals in the scald group had survived. In contrast, only 2 of 30 mice in the scald plus infection group survived for 14 d (6.7% survival). When the infected wounds were treated with NPWT, the survival rate increased significantly to 33.3% (χ^2^ = 5.345, *p* = 0.021). Fewer mice survived when treated with WTD (10%) than with NPWT, and this difference was significant (χ^2^ = 4.286, *p* = 0.038). These results show that NPWT treatment reduced the mortality associated with *P. aeruginosa*–infected burn wounds more than WTD treatment.

### Histology

**Figure 5:**
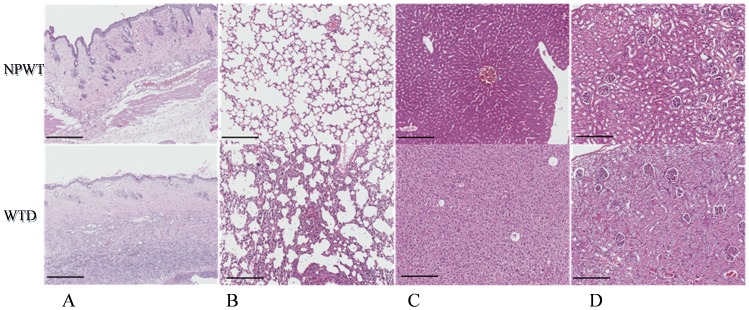
Histopathological examinations following burn wound infection with *P. aeruginosa*. The histopathological analyses included (A) skin within the burn wound, (B) lung, (C) liver, (D) kidney.

Wounded skin, lung, liver, and kidney samples were collected from animals in the NPWT and WTD groups. The tissue sections were examined to determine whether the burn wound infection resulted in systemic defects. Within the wounded skin, fewer leukocytes infiltrated the muscle space in the NPWT group compared with the WTD group (1.41±0.22 *vs*. 3.37±0.54; *t = *15.08, *p*<0.001, Figure 5A). In lung tissue, NPWT significantly reduced the level of thickening associated with the alveolar interstitium, compared with WTD treatment. NPWT also reduced the local lung congestion and leukocyte infiltration (0.94±0.25 *vs*. 2.66±0.49; *t = *13.98, *p*<0.001) during early stages of treatment (Figure 5B). Similar leukocyte infiltration levels were observed in the liver and kidney tissue (liver tissue: 0.20±0.07 *vs*. 0.54±0.11; *t = *11.33, *p*<0.001; kidney tissue: 0.28±0.06 *vs*. 0.47±0.09; *t = *7.92, *p*<0.001). NPWT also decreased the hepatocyte swelling and the number of hepatic cord disruptions (Figure 5C). When the kidney tissues were examined, the mice from the WTD group had more renal tubular epithelial necrosis and renal tubular casts than the NPWT group (Figure 5D). These results suggested that NPWT more effectively alleviated the inflammatory response of the internal organs to the infected burn wound than WTD.

## Discussion

Since its development in 1993 by Fleischman [16], NPWT has been used to treat a wide variety of wounds, including burns, infections, and slow-healing lesions (e.g., bedsores, and diabetic ulcers) [17]–[21]. Many studies have reported that NPWT likely aids wound healing by inhibiting bacterial proliferation within the wound tissue. For example, NPWT effectively cleared bacteria from the wound surface in a porcine model of burned tissue [22]. In clinical cases, NPWT clears Gram-negative bacteria from the wound surface better than traditional change dressings [23]. In Lalliss’s study, *P. aeruginosa* was smeared on the open fracture wounds of goats and the therapeutic effect of NPWT and WTD was compared. The authors found that NPWT more effectively limited *P. aeruginosa* proliferation than WTD [11]. These studies provided preliminary evidence that NPWT inhibits bacterial proliferation. In each case, however, only the wound surface was analyzed. The invasion of pathogenic bacteria into deeper levels of the wound or the diffusion of these bacteria throughout the body has not been examined within the context of NPWT. Moreover, it has not been definitively proven that NPWT can effectively limit the proliferation of *P. aeruginosa* in burn wounds.

To address these issues, we established a murine model of burn-wound sepsis by applying the PAO1 strain (which was engineered to express bacterial luciferase) onto the wound surface [12]. PAO1 is widely used as a test strain to study burn wound infection or sepsis, and its pathogenicity is representative. Many studies have demonstrated that PAO1 was a good test strain for fundamental study in burn wound infection [14], [24], [25]. The mortality rate in this model was over 90% without effective treatment. This result is consistent with our former study [12] and similar to another [14]. In a clinical environment, the mortality of trauma patients with sepsis is as high as 90% [26]; thus, this murine model of burn wound sepsis is to a certain extent consistent with the clinical conditions of burn patients with sepsis.

We then used a small animal imaging system to quantify the amount of fluorescent *P. aeruginosa* on the wound surface as the animals were treated with either NPWT or WTD. During the early stages of infection (i.e., 3 d of treatment), the NPWT group had significantly fewer bacteria in the wound than the WTD group. After 7 d of treatment, however, the bacteria levels were similar in the two groups. This indicates that, for burned tissue, NPWT effectively inhibited the invasion and proliferation of *P. aeruginosa*, whereas WTD treatment did not. Similar results were obtained when the wound secretions were cultured, as the wound bacterial loads were elevated in the WTD group during the first 3 d of treatment (compared with NPWT). These results indicate that NPWT effectively reduced the amount of *P. aeruginosa* in the wound tissue at early stages of burn damage, which agrees with its clinical effects.

Given a burn wound infected with *P. aeruginosa*, we next tested whether NPWT or WTD could prevent pyaemia and protect internal organs from infection. Thus, we examined the serum concentrations of IL-1β, IL-4, IL-6, IL-10, and TNF-α during burn treatment. We also performed histopathological examinations of the wounded skin, lung, liver, and kidney tissue after 3 d of treatment. In the NPWT group, the serum levels of IL-1β, IL-6, and TNF-α did not significantly deviate from baseline throughout the course of the experiment. In contrast, the levels of these proteins in the WTD group were dramatically elevated during the early stages of treatment. On day 7, the bacterial content decreased, which may be related to the mouse immune clearance of the bacteria; a determination of this possibility requires further study. The four cytokines used are Th1-type cytokines, which play important roles in acute post-traumatic inflammation [27]–[29]. Because Th1-type cytokines are primarily secreted by activated monocytes and macrophages, their levels generally correlate with the number of immune cells present in wound tissue or the body [30]. This cytokine analysis indicates, therefore, that NPWT effectively reduces the degree of inflammation by limiting bacterial proliferation and alleviating sepsis progression. Histopathological examinations confirmed these results, as the inflammatory cells in the skin within the burn wound or in the internal organs of the mice in the NPWT group were fewer than those in the WTD group. Inflammation was significantly more extreme in the WTD group. Together, these results suggest that NPWT reduced the *in vivo* inflammatory response and protected internal organs from *P. aeruginosa* infection.

A survival analysis showed that the scald plus infection protocol (without treatment) resulted in a 93.3% mortality rate within 14 d. This is consistent with clinical outcomes that involve burn wounds infected with *P. aeruginosa*, suggesting that we established an effective model of burn-wound sepsis. After the application of either NPWT or WTD treatment, the mortality rates dropped to 66.7% and 90.0%, respectively, and there was a significant difference between the NPWT and WTD groups.

To conclude, NPWT is more effective than WTD in inhibiting the invasion and subsequent proliferation of *P. aeruginosa* into burn-wounded tissue. In addition, NPWT decreased the early mortality rates in our murine model of burn-wound sepsis. The therapeutic effect of NPWT likely results from its ability to inhibit the proliferation of bacteria from the wound surface, which decreases the serum concentrations of Th1-type cytokines and prevents damage to the internal organs. We will continue to use this animal model to characterize the mechanism by which NPWT improves the clinical outcomes associated with skin and soft tissue injuries.

Our study has certain limitations, which include using only WTD as a control treatment. WTD is a basic treatment for burn wounds, and many fundamental studies and clinical investigations have used WTD as a control group [11], [31]–[33]. In addition to WTD, nano-silver dressings, hydrogel dressings, and biological dressings are also widely used in the treatment of burn wounds. Because of the limited experimental groups, we did not compare all of these methods with NPWT in our experiments; these will be included in future studies.
